# Diuretic Response Prediction With MELD Score in Heart Failure

**DOI:** 10.1002/clc.70245

**Published:** 2025-12-26

**Authors:** Volkan Kozluca, Irem Muge Akbulut, Turkan Seda Tan, Halil Gulyigit, Mehmet Emre Ozerdem, Tamer Sayin

**Affiliations:** ^1^ Ankara University Department of Cardiology Ankara Turkey

**Keywords:** cardiorenal, diuresis, MELD, urinary sodium

## Abstract

**Background:**

Heart failure (HF) is still an important disease with high mortality rates worldwide. HF treatment is also difficult due to different phenotypes. Diuretic response (DR) is one of the main differences across these subgroups. Novel urinary parameters are used for DR prediction. We sought to determine whether the MELD score could be used as an additional parameter for predicting the DR.

**Methods:**

Eighty‐one consecutive patients diagnosed with decompensated HF between June and October 2020 were included. The second hour urine sodium (UNa) level after the first intravenous diuretic administration and serum parameters were recorded. All patients underwent a comprehensive echocardiographic examination. MELD score derivatives were tested to assess the DR.

**Results:**

A total of 81 patients (mean age: 66.4 ± 13.5; mean ejection fraction: 29.6 ± 12.7%) were divided into two groups according to UNa. 26 (32%) patients had poor DR. MELD Na score was independently associated with DR (OR = 0.88 [−0.21 to (−0.03)]; *p* = 0.008). Furthermore, MELD Na score was correlated with urinary sodium (*r* = −0.354; *p* = 0.004). Daily furosemide dose was higher (237.9 ± 204.7 vs. 129.3 ± 83.5 mg; *p* = 0.001) and length of hospital stay was longer (15.6 ± 10.8 vs. 8.5 ± 6.1 days; *p* < 0.01) in the low UNa group.

**Conclusion:**

The MELD score derivative was associated with DR according to urinary sodium and may be used as an additional parameter to predict the DR.

**Abbreviations**
CA‐125: cancer antigen 125
DR: diuretic response
EF: ejection fraction
GFR: glomerular filtration rate
HF: heart failure
HFrEF: heart failure with reduced ejection fraction
INR: international normalized ratio
MELD: model for end stage liver disease
NT‐proBNP: n‐terminal pro B‐type Natriuretic Peptide
PASP: systolic pulmonary artery pressure
PoCUS: point‐of‐care ultrasound
RAAS: renin‐angiotensin‐aldosterone system
TAPSE: tricuspid annular plane systolic excursion
TTE: transthoracic echocardiography
UNa: urinary sodium
VeXUS: venous excess ultrasound

## 1 Introduction

Heart failure (HF) is a major cause of morbidity and mortality. The global burden of HF is rising due to an aging population [1]. There are different phenotypes in HF. Ejection fraction (EF) is mostly used for classifying patients. Phenotyping HF is a dynamic process according to treatment (improved EF) or definition updates (midrange‐mildly reduced EF) [2]. HF with preserved EF also has its own subphenotypes such as pulmonary vascular disease and right HF. Treatment response and prognosis of these phenotypes are different [3].

Diuretic resistance and adverse renal outcomes are more common in patients with right HF [4, 5]. Although there is no precise definition of diuretic resistance, some parameters such as spot urinary sodium can be used to detect treatment response [6].

Some traditional biomarkers are used for prognosis like serum sodium [7], serum bilirubin [8], glomerular filtration rate, but somehow combination of these parameters in a validated score can predict clinical outcomes more precisely. Although the Model for End‐stage Liver Disease (MELD) score was mainly developed for patients with end‐stage liver disease [9], it can be used as an index for backward HF.

This study aimed to evaluate whether poor diuretic response (DR) according to urinary sodium is associated with MELD score. In addition, we sought to determine the correlation between DR and MELD score derivatives. We hypothesized that if MELD is highly associated with DR, the score may add incremental value to DR prediction.

## 2 Materials and Methods

### 2.1 Patient Data

In a retrospective and single‐center study, 81 patients admitted between June and October 2020 were screened for analysis. HF decompensation was diagnosed based on specific symptoms, signs, biomarkers, and imaging according to the European Society of Cardiology HF Guidelines (2021). The Ethics Committee of Ankara University approved the study design. Warfarin users and patients with end‐stage liver disease were excluded from the study. Baseline characteristics and echocardiographic measurements were recorded.

### 2.2 Diuretic Response

Diuretic therapy is the mainstay of decongestion in HF. Diuretic agents mainly target the renal tubulary system via electrolyte pumps (mainly sodium exchange or secretion) and cause sodium and water diuresis. DR is calculated with various parameters like urine output, weight change, and, in recent years, a more quantitative marker, spot urine sodium (UNa) concentration. UNa is recommended for rapid DR prediction in the European Society of Cardiology HF guideline [10]. If UNa is lower than 50 mEq/L in the second hour after the first intravenous diuretic administration, poor DR is predicted for the patient. On the other hand, UNa greater than 70 mEq/L indicates a good response. In definition, diuretic resistance is failure to increase fluid and sodium (Na+) output sufficiently despite escalating therapeutic agent doses. Diuretic resistance is a consequence of renin‐angiotensin‐aldosterone system activation and can be managed by optimizing the dose and timing of loop diuretics, combining different classes of diuretics that act on different nephron segments, reducing salt intake, correcting electrolyte abnormalities, and using other drugs that improve cardiac function or reduce neurohormonal activation [11, 12].

The exact prevalence of diuretic resistance is difficult to determine because there is no standard definition or diagnostic criteria for this condition. However, some studies have estimated that 20%–30% of patients with HF have diuretic resistance. This may vary depending on the severity of HF, the type and dose of diuretics used, and the presence of other factors that affect the DR. Diuretic resistance is associated with worse outcomes, such as higher mortality and rehospitalization rate [11, 12].

### 2.3 MELD Scoring System

MELD is a biomarker scoring system for assessing chronic liver disease severity. Score was developed to predict outcomes in patients who had undergone transjugular intrahepatic portosystemic shunt procedure (TIPS) [13]. It was then found to be useful in determining prognosis in liver transplant candidates [14]. The original MELD score is described with serum bilirubin, international normalized ratio for prothrombin time (INR), and serum creatinine. The MELD score evolved in the meantime, and the etiology of liver disease was excluded from the score. The United Network for Organ Sharing (UNOS) has made some modifications in the score by adding recent dialysis factor and serum sodium (MELD‐Na). The MELD‐XI score (score without INR) was derived from the original score for patients on anticoagulant therapy [15]. End‐stage liver disease and HF have common pathways in splanchnic and renal haemodynamics. Because of these common pathways, MELD has become a measure of cardiorenal stress. In our study, MELD score derivatives were calculated according to data on admission day and tested in different DR groups. Patients using anticoagulation or those with preexisting end‐stage liver disease were excluded from the study because of interference with MELD calculation. MELD score derivatives and formulas are shown in Table 1.

**Table 1:** Calculation of MELD score derivatives.

Score	Components
MELD (Pre‐2016/Original)	9.6 * log_e_ (creatinine mg/dL) + 3.8 * log_e_ (bilirubin mg/dL) + 11.2 * log_e_ (INR) + 6.4
MELD‐Na (sodium)	MELD + 1.59 * (135 – Na(mEq/L))
MELD‐XI	5.11 * log_e_ (bilirubin mg/dL) + 11.76 * log_e_ (creatinine mg/dL) + 9.44

### 2.4 Echocardiography

Eighty‐one patients who met the inclusion criteria for the study underwent echocardiographic examination at Ankara University Heart Center. An experienced cardiologist performed TTE using a Vivid E9 imaging system (GE Medical Systems, Chicago, USA), and measurements were obtained according to the recommendation of the American Society of Echocardiography Guidelines. The left ventricular EF was calculated from 4‐chamber and 2‐chamber views with the modified Simpson method. TAPSE was calculated from apical 4‐chamber views with M‐mode imaging. Systolic pulmonary artery pressure (PASP) was calculated using tricuspid regurgitation velocity and inferior vena cavae collapsibility.

### 2.5 Statistical Analysis

Quantitative variables were specified as mean ± standard deviation, categorical variables were specified as frequency and percentage. All patients were divided into two groups based on DR according to spot UNa within the second hour after the first intravenous diuretic administration. Baseline characteristics were compared with Student‐*T* tests for continuous variables and chi‐square tests for categorical variables. Kruskal−Wallis test was used for variance analysis of nonparametric variables. Statistically significant parameters were also assessed with regression analysis. Spearman or Pearson regression analysis was used for the correlation between MELD derivatives and DR. Cutoff values were tested with ROC curve analysis to predict DR. Statistical analyzes were conducted with the Jeffreys's Amazing Statistics Program (JASP) software version 0.19.1.0. Significance level was 0.05 for *p* values.

## 3 Results

### 3.1 Baseline Characteristics

The study population included a total of 81 patients (mean age: 66.4 ± 13.5; female gender: 37%) with decompensated HF. Patients were divided into two groups according to spot UNa levels. The poor DR was seen in 32% of the patient population. Demographic and clinical characteristics, biomarkers, and echocardiographic parameters were compared between the groups. Mean MELD Na score was 18.6 ± 6.8 in all patients, and mean EF was 29.6% ± 12.7%. Right ventricular systolic function (mean TAPSE: 13 ± 3.6 mm, range: 7–20 mm) was depressed in 46 (58%) patients, and haemodynamic congestion biomarker (mean NT‐proBNP: 9012 ± 9190 pg/mL, range: 306–35 000 pg/mL) was increased in all study population. On the other hand, DR indicators, including daily diuretic dose and worsening renal function prevalence were different between the groups. Baseline characteristics are shown in Table 2.

**Table 2:** Baseline characteristics.

Characteristic (No. of patients %) (*n* = 81)	
Age	66 ± 13
Female gender	30 (37%)
Ischemic etiology	47 (58%)
Diabetes	44 (54%)
Atrial Fibrillation	34 (42%)
Medications (No. of patients %)	
Beta blocker	74 (91%)
ACEi or ARB	50 (61%)
MRA	71 (87%)
SGLT2i	11 (13%)
Biomarkers (mean ± SD)	
GFR (mL/min)	56.4 ± 23.8
NT‐proBNP (pg/mL)	9012 ± 9190
Urinary Sodium (meq/L)	73.9 ± 37.8
MELD‐Na	18.6 ± 6.8
MELD XI	26 ± 7.3
Echocardiographic parameters (mean ± SD)	
Ejection Fraction (%)	29.6 ± 12.7
TAPSE (mm)	13.1 ± 3.6
PASP (mmHg)	55 ± 12.4
TAPSE/PASP ratio	0.24 ± 0.08

Abbreviations: BUN, blood urea nitrogen; GFR, glomerular filtration rate; INR, international normalized ratio; NT‐proBNP, N‐terminal pro‐B‐type natriuretic peptide; PASP, systolic pulmonary artery pressure; TAPSE, tricuspid annular plane systolic excursion; WRF, worsening renal function.

### 3.2 Echocardiographic Findings

The mean EF was 29.6% ± 12.7%, and the mean TAPSE was 13 ± 3.6 mm. There was no difference in EF and TAPSE between the groups (*p* = 0.509 and *p* = 0.104). PASP and TAPSE/PASP ratio, a noninvasive right ventricle‐pulmonary artery coupling parameter, were also similar between groups (*p* = 0.59, and *p* = 0.067).

### 3.3 Diuretic Response Parameters

The basal glomerular filtration rate was similar and low GFR prevalence was not different between the groups. However, daily furosemide dose was statistically higher (237.9 ± 204.7 vs. 129.3 ± 83.5 mg, *p* = 0.001) in the poor response group and length of hospital stay was longer (15.6 ± 10.8 vs. 8.5 ± 6.1 day, *p* < 0.001). Diuretic resistance indicator such as worsening renal function rate was higher (*p* < 0.001) in the low UNa group but ultrafiltration rate and need for inotropic support were similar in the groups.

### 3.4 Laboratory Results

Laboratory results on admission day were recorded. Serum blood urea nitrogen, serum creatinine, serum albumin, and total bilirubin levels were similar in the groups. Serum sodium was statistically lower (*p* = 0.03) and INR was higher (*p* = 0.02) in the poor response group. NT‐proBNP was used as a hemodynamic congestion marker and the poor response group had a higher NP level trend but there was no statistical difference between groups (10267 ± 10741 vs. 8419 ± 8401 pg/mL, *p* = 0.402).

### 3.5 Meld Score

MELD score derivatives were calculated using existing formulas (Table 1) with admission day parameters. Original MELD score was not different between groups (*p* = 0.18). MELD XI score tended to be higher in the low UNa group but did not reach statistical significance (*p* = 0.057).

We were able to calculate the MELD Na score in 66 patients among the study population, and the score was statistically higher in the poor response group (*p* < 0.01). MELD Na score was also correlated with DR according to spot UNa at the second hour (*p* < 0.01). With ROC curve analysis, a cut‐off value greater than 14.5 predicts poor DR (AUC = 0.7; 92% sensitivity, %63 specificity, *p* = 0.007). Results according to subgroups are shown in Table 3.

**Table 3:** Results of Subgroups.

Characteristics	Poor DR group (UNa < 50 mEq/L) (*n* = 26)	Good DR group (UNa > 70 mEq/L) (*n* = 55)	*p* value
Age (years)	62 ± 13	68 ± 13	*p* = 0.063
Female gender (no. of patients %)	9 (34%)	21 (38%)	*p* = 0.75
Diabetes (no. of patients %)	18 (69%)	26 (47%)	*p* = 0.09
Laboratory results			
GFR (mL/min)	54.1 ± 22.1	57.5 ± 24.7	*p* = 0.56
Low GFR (< 60 mL/min) prevalence (%)	15 (57%)	30 (54%)	*p* = 0.79
Serum BUN (mg/dL)	37.7 ± 18.7	32.5 ± 14.7	*p* = 0.18
Serum creatinine (mg/dL)	1.44 ± 0.55	1.34 ± 0.54	*p* = 0.47
Serum sodium (mEq/L)	134.8 ± 5.1	137.4 ± 4.7	*p* = 0.03
Serum albumin (mg/dL)	35.8 ± 4.1	37 ± 5	*p* = 0.31
INR	2.4 ± 2.1	1.5 ± 0.8	*p* = 0.02
Bilirubin total (mg/dL)	1.69 ± 1.38	1.23 ± 0.75	*p* = 0.08
NT‐proBNP (pg/mL)	10267 ± 10741	8419 ± 8401	*p* = 0.402
Echocardiographic parameters			
Ejection fraction (%)	28.2 ± 13.3	30.2 ± 12.5	*p* = 0.509
TAPSE (mm)	12.1 ± 3.9	13.6 ± 3.4	*p* = 0.104
PASP (mmHg)	56.1 ± 12.9	54.5 ± 12.2	*p* = 0.59
TAPSE/PASP ratio	0.22 ± 0.09	0.26 ± 0.08	*p* = 0.067
Diuretic response characteristics			
Length of stay (day)	15.6 ± 10.8	8.5 ± 6.1	*p* < 0.001
Daily furosemide dose (mg)	237.9 ± 204.7	129.3 ± 83.5	*p* = 0.001
WRF (No. of patients %)	18 (69%)	13 (23%)	*p* < 0.001
Inotropic support (No. of patients %)	6 (23%)	10 (18%)	*p* = 0.605
Ultrafiltration need (No. of patients %)	1 (3%)	1 (1.8%)	NA
Urine sodium (mg/dL)	28.1 ± 11.3	95.5 ± 24.1	*p* < 0.001
MELD score derivatives			
MELD Na (mean ± SD)	21.6 ± 7.4	16.7 ± 5.7	*p* = 0.004
MELD Na (median and IQR)	20 (8)	16 (8)	*p* < 0.01
MELD XI (mean ± SD)	28.2 ± 7.1	24.7 ± 7.2	*p* = 0.057
MELD Pre‐2016 (mean ± SD)	15.7 ± 6.1	13.9 ± 5.3	*p* = 0.18

### 3.6 Correlation and Binary Logistic Regression Analysis of MELD Derivatives

We tested different derivatives of the MELD score in predicting DR using binary logistic regression analysis. MELD Na score was a significant predictor of DR (OR: 0.88 β (95% CI) (−0.21 to [−0.03]), *p* = 0.08). Original MELD and MELD XI scores did not reach statistical significance (*p* = 0.18 and *p* = 0.06, respectively).

**Figure 1:**
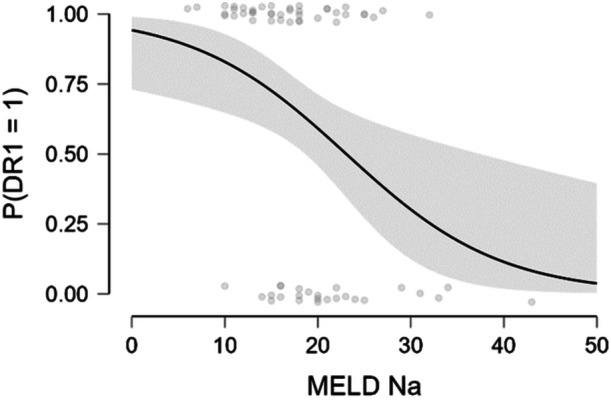
Correlation between MELD Na score and diuretic response (*r* = −0.354, *p* = 0.004) (DR1: diuretic response, DR1 = 1 indicates good diuretic response).

The Pearson method was used to assess the correlation between MELD parameters and DR (Table 4). Although there was a weak correlation between serum sodium and INR, a moderate correlation was observed between MELD Na score and DR (Figure 1). There was no correlation between serum creatinine or total bilirubin.

**Table 4:** Correlation between diuretic response and MELD parameters.

Variable	*R*	*p* value
Creatinine	−0.115	0.31
Sodium	0.237	0.03
INR	−0.266	0.02
Total bilirubin	−0.212	0.08
MELD Na	−0.354	0.004

MELD score parameters were also analyzed with a univariate logistic regression model. In statistical modelling, lower serum sodium {OR: 1.113 β (95% CI) (0.004–0.21), *p* = 0.04} and higher INR values (OR: 0.68 β (95% CI) [−0.73 to (−0.026)], *p* = 0.035) were significant predictors of poor DR.

## 4 Discussion

Diuretic resistance in HF is independently related to worse outcomes and prognosis. This study demonstrated that the MELD Na score was correlated with DR, and higher scores had an independent relationship with poor DR.

MELD was investigated for HF in previous studies [16, 17, 18, 19], and a high score was associated with poor prognosis. In our analysis, MELD Na score was higher in the poor prognostic group according to DR. Diuretic resistance or cardiorenal stress may be the key component in this prognostic connection. Kim et al. showed that MELD serum parameters (Serum sodium, INR, and total bilirubin) were also prognostic indicators of HF. In our study, MELD parameters, except for total bilirubin, were consistent with this study. The score was mainly an indicator of portal hypertension grade in end‐stage liver disease, but it is useful and effective because of its capability to analyze multiorgan function. As expected, serum sodium, an indicator of RAAS activation, and INR, an indicator of liver congestion, were statistically significant parameters in the MELD score. Removing a parameter from the score reduces its statistical significance, unlike in previous studies [16, 19]. In particular, the MELD Na derivative was statistically more precise than its confounding parameters. Palazzuoli et al. [20] studied different DR groups in acute decompensated HF patients and found that glomerular function parameters (GFR, serum creatinine) were similar in the groups. We also observed no difference in GFR, low GFR prevalence, and serum creatinine between the groups. As far as we know, no previous research has investigated the correlation between the MELD score and DR according to UNa.

Although the DR was different between groups, similar echocardiographic indicators (EF, TAPSE) were observed and consistent with the study of Cobo‐Marcos et al. [21]. This finding indicates that haemodynamic inferences should be made for predicting DR, apart from the parameters that we traditionally evaluate. In this sense, the use of new biomarkers (like CA‐125) or imaging scores (like PoCUS; VeXUS grade) is increasing for clinical evaluation of cardiorenal interaction or splanchnic congestion.

## 5 Limitation

Our sample size is relatively small because it was a retrospective and single‐center study. We included mostly HFrEF patients with right ventricular dysfunction. We used indirect estimate parameters for systolic function and PASP values. Further research is needed to validate the MELD score with its dynamic variability with a large number of patients and to present the mechanisms for DR.

## 6 Conclusion

In conclusion, our study is remarkable for indicating the relationship between MELD score and DR. We also demonstrated that a high MELD score was associated with a higher daily furosemide dose, longer hospital stay, and more frequent worsening renal function. We also defined MELD as an index for cardiorenal interaction and a marker of a HF phenotype. A schematic overview of the study concept and clinical workflow is provided in the Central Illustration 1.

**Central illustration 1:**
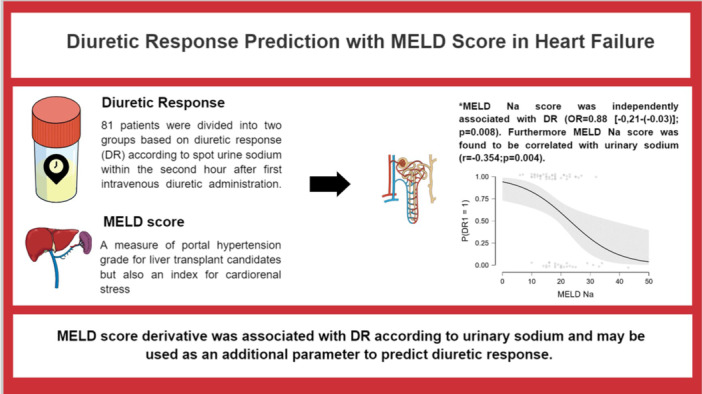
Diuretic response prediction with MELD score in heart failure.

## Funding

The authors received no specific funding for this work.

## Consent

The authors confirm that patient consent is not applicable to this article because this is a retrospective analysis using de‐identified data.

## Conflicts of Interest

The authors declare no conflicts of interest.

## Data Availability

The data that support the findings of this study are available from the corresponding author upon reasonable request.
